# Molecular characterization and pathogenicity of an infectious clone of tomato leaf curl New Delhi virus isolate infecting *Cucumis melo*

**DOI:** 10.1007/s44154-023-00128-8

**Published:** 2023-11-23

**Authors:** Yuzhen Mei, Lingmin Cai, Yaqin Wang, Fangfang Li, Xiuling Yang, Jinghua Yang, Xueping Zhou

**Affiliations:** 1 State Key Laboratory of Rice Biology, Institute of Biotechnology, Zhejiang University, Hangzhou, 310058 Zhejiang China; 2 State Key Laboratory for Biology of Plant Diseases and Insect Pests, Institute of Plant Protection, Chinese Academy of Agricultural Sciences, Beijing, 100193 China; 3 Laboratory of Germplasm Innovation and Molecular Breeding, Institute of Vegetable Science, Zhejiang University, Hangzhou, 310058 China; 4 Yazhou Bay Science and Technology City, Hainan Institute, Zhejiang University, Sanya, 572025 China

**Keywords:** Begomovirus, Tomato leaf curl New Delhi virus, Infectious clone

## Abstract

Tomato leaf curl New Delhi virus (ToLCNDV) is a member of the genus *Begomovirus*, and causes devastating disease in the world. In recent years, ToLCNDV was rapidly spreading in China and induces severe economic losses in agriculture. In this study, we sequenced and characterized the complete genome of ToLCNDV isolates from melon plants showing leaf curling and stunting symptoms in Jiangsu Province of China. We constructed a full-length infectious cDNA clone of ToLCNDV, which could induce systemic infection with typical symptoms in *Nicotiana benthamiana*, *Citrullus melo*, and *Citrullus lanatus* plants through agrobacterium-mediated inoculation. Further experimental evidence demonstrated that the virions produced in plants infected with the infectious clone of ToLCNDV are biologically active and sap-transmissible. We also evaluated the resistance of commercial melon cultivars to ToLCNDV and found all testing melon cultivars were susceptible to ToLCNDV. Collectively, the reverse genetic system developed herein will facilitate further research on biological functions of proteins encoded by ToLCNDV and plant-ToLCNDV interactions, which might provide new insights into breeding resistance germplasm in crops.

**Supplementary Information:**

The online version contains supplementary material available at 10.1007/s44154-023-00128-8.

## Introduction

The *Geminiviridae* family contains a group of single-stranded DNA (ssDNA) viruses encapsidated within geminate particles that cause devastating diseases in many crops around the world (Boulton et al. 2003; Rojas et al. 2005; Mansoor et al. 2006; Fauquet et al. 2008; Navas-Castillo et al., 2011; Sattar et al. 2013; Yang et al. 2019; Medina-Puche et al. 2022). The *Geminiviridae* is divided into 14 genera (*Becurtovirus*, *Begomovirus*, *Capulavirus*, *Citodlavirus*, *Curtovirus*, *Eragrovirus*, *Grablovirus*, *Maldovirus*, *Mastrevirus*, *Mulcrilevirus*, *Opunvirus*, *Topilevirus*, *Topocuvirus*, and *Turncurtovirus*) on the basis of host range, genome structure, and insect vector (Roumagnac et al. 2022). The genus *Begomovirus* contains 445 species that have either one (monopartite) or two (bipartite) genomic components and is the largest genus of the *Geminiviridae* family (Harrison & Robinson 1999; Hanley-Bowdoin et al. 2013). Bipartite begomoviruses consist of DNA A and DNA B components that encoded six proteins (AC1, AC2, AC3, AC4, AV1, AV2) and two proteins (BC1 and BV1), respectively. The DNA A component encodes proteins involved in viral replication, transcription, and virion assembly, whereas the DNA B component encodes proteins required for virus movement.

Tomato leaf curl New Delhi virus (ToLCNDV) is one of the typical bipartite begomoviruses, which was first reported in India (Srivastava et al. 1995) and was recently spreading in China (Li et al. 2022; Zeng et al. 2023; Gu et al. 2023). ToLCNDV is naturally transmitted by whiteflies and infects a wide range of host plants belonging to 17 families, such as *Solanaceae*, *Cucurbitaceae*, and *Fabaceae*. Infection by ToLCNDV often causes leaf curling, vein swelling, and stunting symptoms in plants (Zaidi et al. 2016; Zaidi et al. 2017). The full-length genomic sequences of ToLCNDV are genetically variable and some of the ToLCNDV strains were reported to be sap transmissible whereas some are not (Tsai et al. 2011; Lopez et al. 2015).

Planting resistant varieties is an effective way to control geminiviral diseases, however, only a few genes were reported to confer plant resistance to ToLCNDV. Pyramiding *Ty-2* and *Ty-3* tomato lines exhibited a high level of resistance to ToLCNDV (Prasanna et al. 2015). *SlSw5a*, a *R* gene cloned from a ToLCNDV-resistant tomato cultivar that lacks the known *Ty* genes confers resistance to ToLCNDV by interacting with ToLCNDV AC4 and triggering hypersensitive response (HR) at infection sites to limit the spread of the virus (Sharma et al. 2021).

Infectious clones of plant viruses are important tools for the investigation of virus pathogenicity and virus-host interactions as well as screening of resistant plant cultivars. Here, we characterized the complete genome sequence of a ToLCNDV isolate (named ToLCNDV-JS1) infecting melon (*Cucumis melo*) in Jiangsu Province of China, and constructed an infectious clone of ToLCNDV-JS1 that shows infectivity in *Nicotiana benthamiana* and melon plants. Moreover, we evaluated the resistance of melon cultivars through agroinoculation of the infectious clone of ToLCNDV-JS1. The infectious clone constructed in this study would be useful to study the pathogenicity of ToLCDNV and identify resistance germplasm in *Cucurbit*s and other crops.

## Results

### Symptom observation and detection of ToLCNDV in melon

**Fig. 1:**
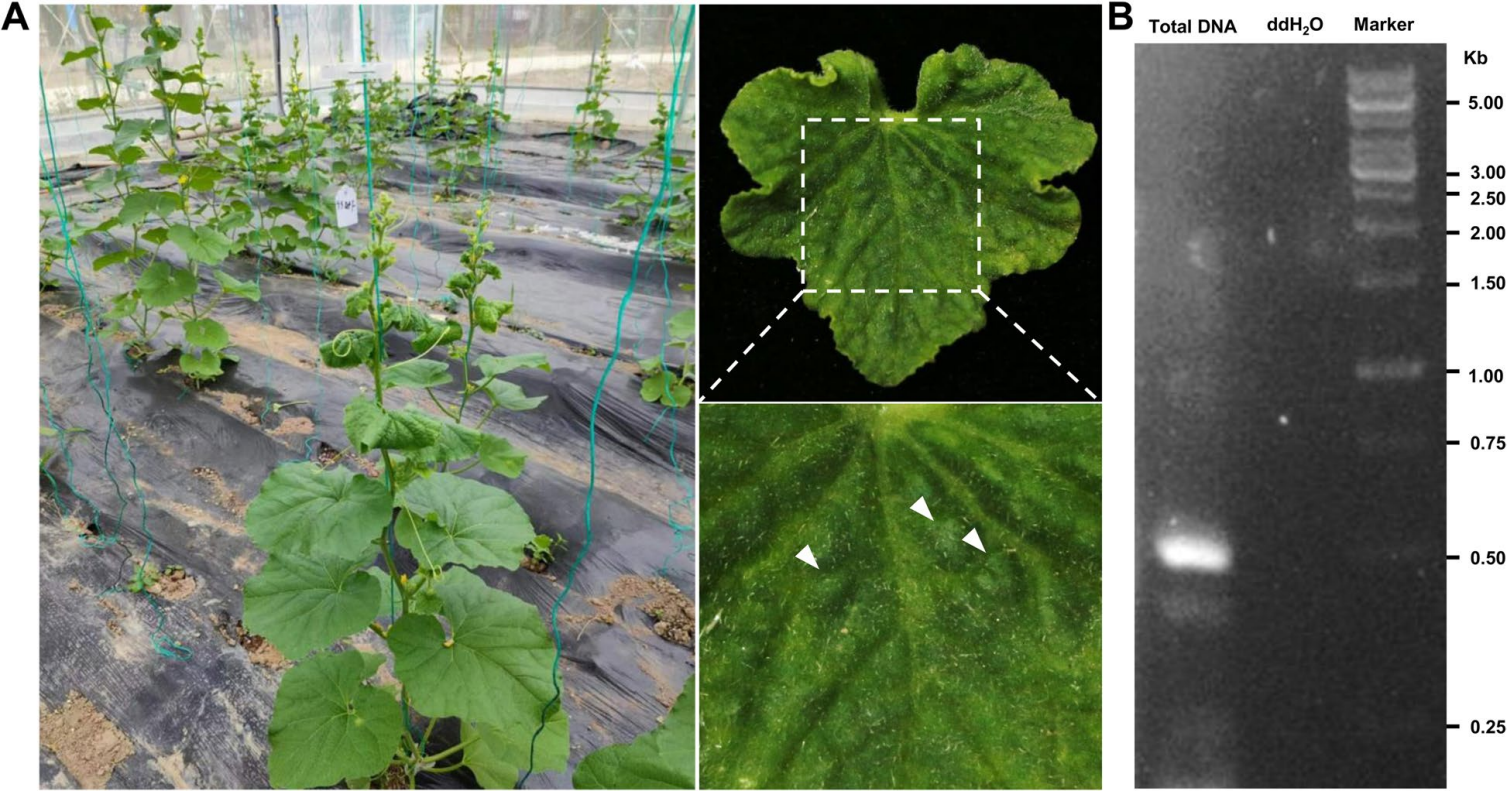
Detection of tomato leaf curl New Delhi virus (ToLCNDV) from symptomatic melon leaves. **A** Downward leaf curling symptoms on the younger leaves of melon in a greenhouse in Jiangsu Province, China. Characteristic symptoms in the leaf are cropped and zoomed in the right panel. White arrows indicate callus-like tissues in a melon leaf. **B** Detection of ToLCNDV by PCR with the primer pair PA/PB. Lane 1 indicates total DNA extracted from symptomatic leaves shown in (**A**) was used as the template of PCR amplification; lane 2, double distilled water (ddH2O) was used as a negative control for PCR amplification

During a survey of melon-infecting viruses, melon plants showing leaf curling and stunting symptoms were found in greenhouses in Nantong of Jiangsu Province of China in October 2022 (Fig. 1A). To determine whether the symptomatic melon plants were infected by begomoviruses, symptomatic leaves were collected from four melon plants, and total DNA was extracted, and the degenerate primer pair PA/PB that is expected to amplify an approximately 500 bp-fragment covering part of the intergenic region (IR) and the *AV2* gene of begomoviral DNA A was used to identify potential begomoviruses. PCR results showed that a 500 bp fragment could be amplified from the total DNA extracted from the tested diseased melon leaves (Fig. 1B). The PCR products were cloned and sequenced, and the 500-bp sequence was used as the query sequence to search NCBI database. BLASTN analysis showed that this sequence has a high similarity with ToLCNDV DNA A (OQ190945.1), suggesting that the observed symptoms might be caused by ToLCNDV.

### Characterization of the ToLCNDV genome and affinities to other begomoviruses

**Fig. 2:**
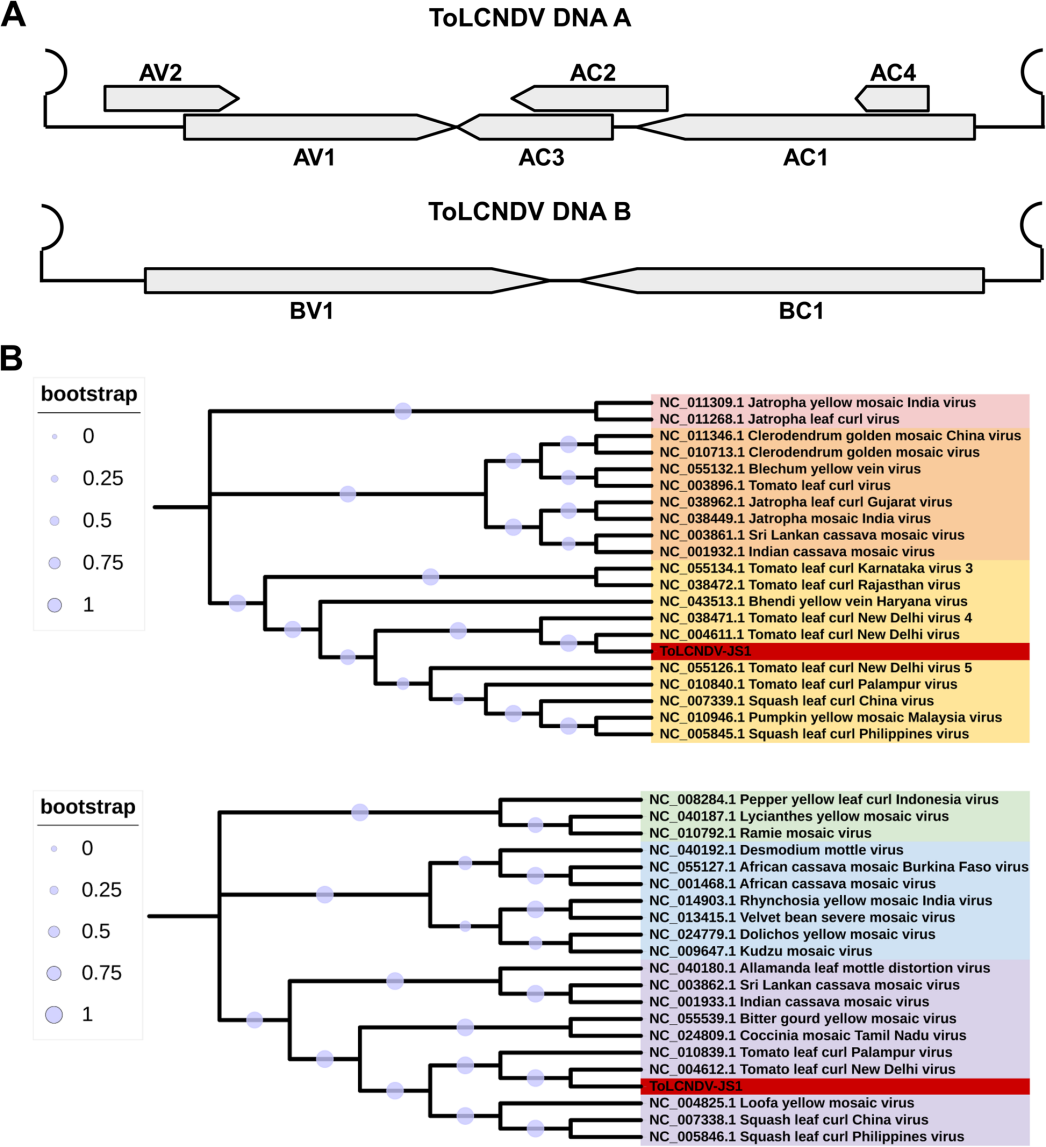
Genome characterization and phylogenetic analysis of the DNA A and DNA B component of ToLCNDV. **A** Schematic representation of the linearized genomic structue of ToLCNDV-JS1. The gray arrows indicate the open reading frames of ToLCNDV DNA A and DNA B. **B** Phylogenetic analysis of the complete genome sequence of the DNA A (upper panel) and DNA B (lower panel) component of ToLCNDV-JS1 with other selected begomoviruses. The scale bar representing the genetic distance of different begomoviruses is shown as indicated

The complete nucleotide sequences of DNA A and DNA B of a ToLCNDV Jiangsu isolate (ToLCNDV-JS1) were determined to be 2739 nt and 2692 nt (GenBank accession numbers OR157979 and OR157980), respectively, containing eight open reading frames (AC1, AC2, AC3, AC4, AV1, AV2, BC1, and BV1) (Fig. 2A). To elucidate the evolutionary relationship of ToLCNDV-JS1 with other begomoviruses, phylogenetic analysis was performed using the complete genome sequences of ToLCDNV and other selected begomoviruses. ToLCNDV-JS1 DNA A was grouped into the same clade with other ToLCNDV isolates, sharing the highest nucleotide identity (99.74%) with the DNA A sequence of a ToLCNDV isolate infecting tomato in China (OQ190945.1). ToLCNDV- JS1 DNA B was grouped in the same cluster with other ToLCNDV isolates with the closest relatives of the DNA B of two ToLCNDV isolates from China (OQ184747.1 and OP683996) (Fig. 2B).

### Infectivity and pathogenicity of the infectious clone of ToLCNDV-JS1

**Fig. 3:**
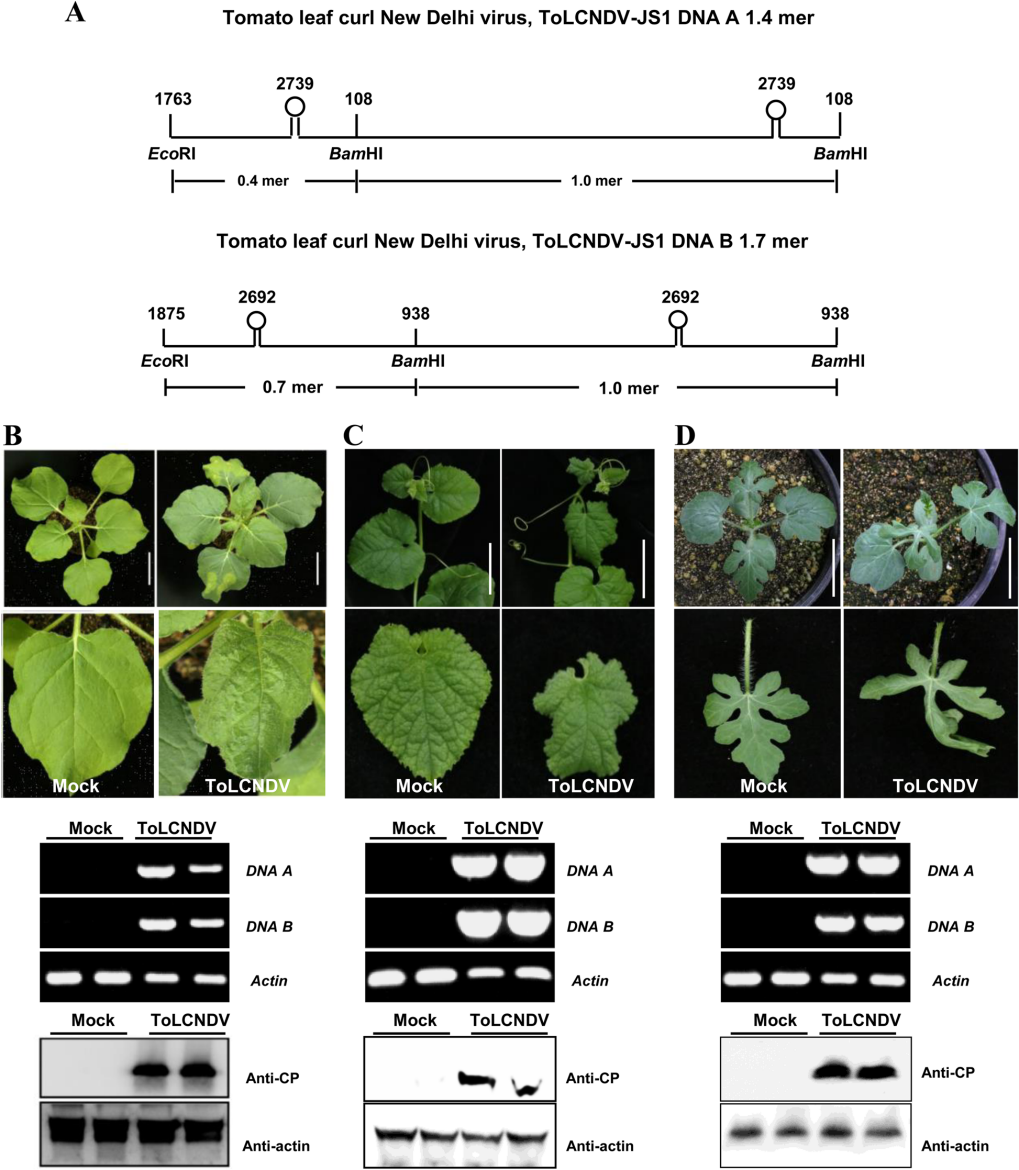
Infectivity and pathogenicity of the infectious clone of ToLCNDV-JS1. **A** Strategies for construction of the infectious clones of ToLNDV-JS1. 1.4-mer and 1.7-mer tandem repeats of ToLCNDV DNA A and DNA B were constructed to the plant binary vector pBinPLUS, respectively. The restriction enzymes used for the construction of the infectious clone of ToLCDNV-JS1 were shown as indicated. **B**-**D** Analysis of the infectivity and pathogenicity of ToLCNDV infectious clone. Symptoms induced by ToLCNDV-JS1 in *Nicotiana benthamiana* at 6 dpi (**B**), *Citrullus melo* at 30 dpi (**C**) and *Citrullus lanatus* at 16 dpi (**D**). Tested plants were inoculated with equal volume of agrobacterium cultures harboring the infectious clone of DNA A and DNA B of ToLCNDV, respectively. Bars = 5 cm. PCR detection of ToLCNDV viral DNA using total DNA extracted from mock or ToLCDNV-infected plants as indicated. The *Actin* genes from *N. benthamiana*, *Citrullus melo* and *Citrullus lanatus* plants were used as internal controls. Western blot analysis of ToLCNDV infection in systemic leaves of agroinoculated plants using the antibody raised against coat protein (CP). The membrane stripped and probed with the antibody against actin was used as the loading control

To identify the pathogenicity of ToLCNDV-JS1, an infectious clone of ToLCNDV-JS1 was constructed. The 1.4-mer and 1.7-mer tandem repeats of full-length genomic sequences of DNA A and DNA B were cloned into the binary vector pBinplus, respectively (Fig. 3A). The infectivity and pathogenicity of the infectious clone of ToLCNDV-JS1 was tested in *Nicotiana benthamiana*, melon, and watermelon (*Citrullus lanatus*), respectively. Plants agroinoculated with the empty vector were used as mock plants. Leaf curling symptoms were observed in the newly emerging leaves of *N. benthamiana* inoculated with the infectious clone of ToLCDNV at 6 days post-inoculation (dpi) (Fig. 3B). Leaf curling symptoms were observed in melon (Fig. 3C) and watermelon plants (Fig. 3D) at 30 dpi and 16 dpi, respectively. PCR amplification of viral DNA using ToLCNDV-specific primers showed that both the DNA A and DNA B component of ToLCNDV were present in *N. benthamiana* and tested *Cucurbitaceae* plants agroinoculated with the infectious clone of ToLCNDV but not in mock plants. Western blot analysis using the antibody raised against coat protein (CP) of ToLCNDV by the authors lab further demonstrated the accumulation of ToLCNDV in all the three tested plants inoculated with the infectious clone of ToLCNDV-JS1 (Fig. 3B-D). These results suggested that the infectious clone of ToLCNDV-JS1 constructed in this study is infectious and could induce typical symptoms in *N. benthamiana*, melon, and watermelon plants.

### Sap transmission of the infectious clone of ToLCNDV-JS1

**Fig. 4:**
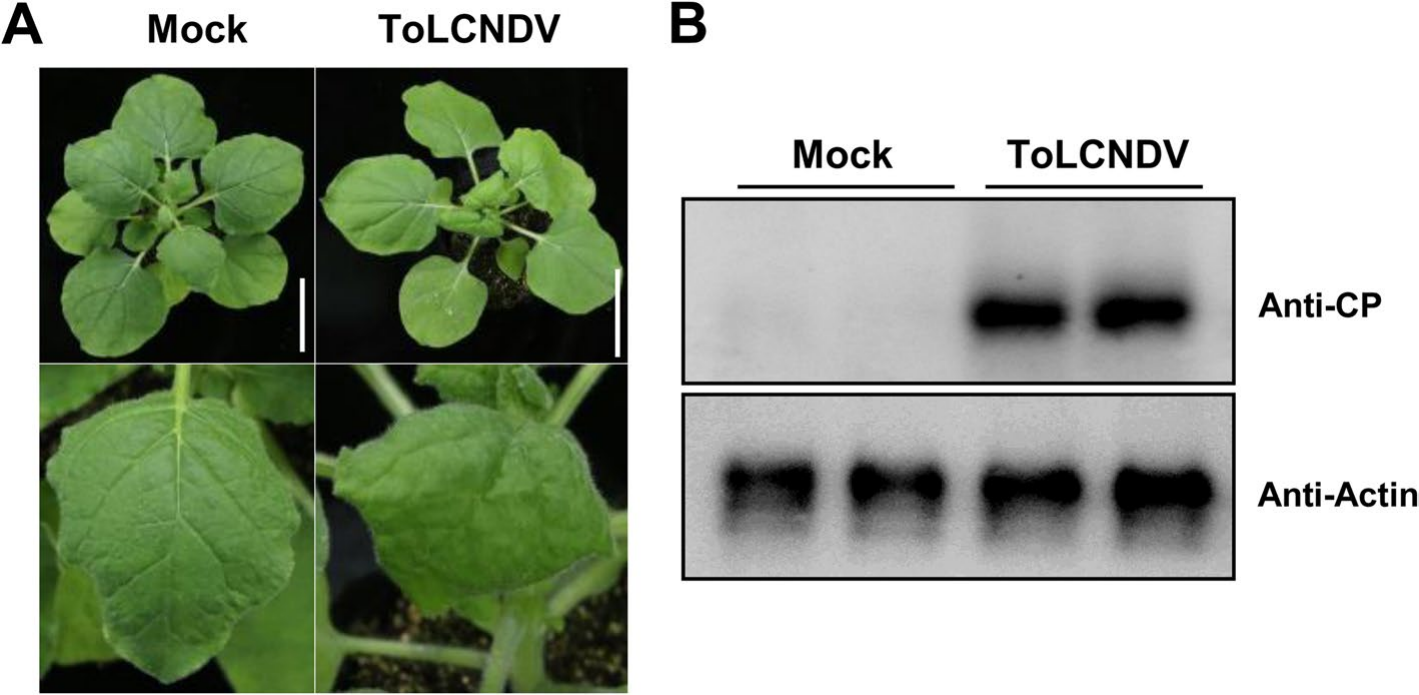
Sap transmission of the infectious clones of ToLCNDV-JS1. **a** Downward leaf curing symptoms in *Nicotiana benthamiana* plants mechanically inoculated with the crude sap extracted from systemic leaves of melon plants infected with the infectious clone of ToLCNDV. *N. benthamiana* plants mechanically inoculated with the crude sap extracted from healthy melon leaves were used as mock. Photographs were taken at 9 dpi. **b** Western blot analysis of ToLCNDV in systemic leaves of mechanically inoculated *N. benthamiana* plants indicated in (a). ToLCNDV was detected using an antibody raised against the coat protein (CP) and actin was used as the loading control

To assess whether ToLCNDV-JS1 is mechanically transmissible, the crude sap extracted from the symptomatic leaves of melon plants agroinoculated with ToLCNDV infectious clone (ToLCDNV sap) was gently rubbed onto the leaves of healthy *N. benthamiana* plants. The crude sap extracted from leaves of healthy melon plants was used as mock inoculum. Compared to the *N. benthamiana* plants inoculated with the mock inoculum, the systemic leaves of ToLCNDV sap-inoculated *N. benthamiana* plants displayed leaf curling symptoms indistinguishable from those agroinoculated with the infectious clone of ToLCNDV (Fig. 4A). As expected, western blot analysis showed that ToLCNDV was indeed detected in the systemic leaves of *N. benthamiana* plants mechanically inoculated with ToLCNDV sap (Fig. 4B). These results suggested that viral progeny from agroinoculated melon plants was readily mechanically transmissible to *N. benthamiana* plants.

### Evaluation of the resistance of commercial melon cultivars to ToLCNDV

**Fig. 5:**
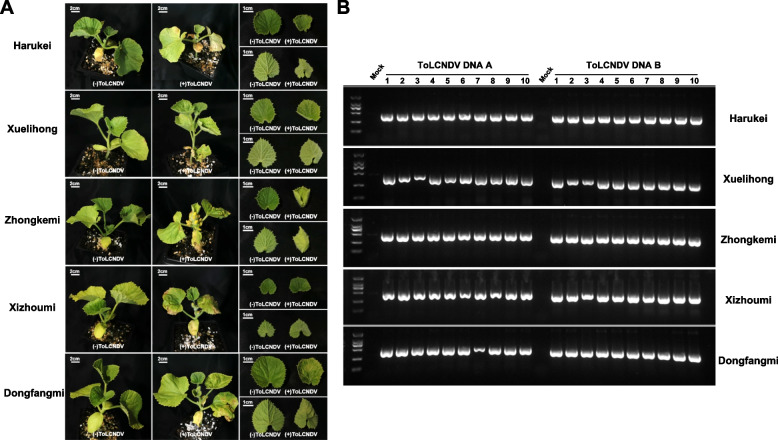
Susceptibility of commercial melon cultivars to ToLCNDV. **A** Symptoms induced by ToLCNDV**-**JS1 in commercial melon cultivars. The melon cultivars of melon used for inoculation with the infectious clone of ToLCNDV-JS1 or the empty vector pBinPLUS were indicated leaf curling symptoms were shown in the right panels. Ten plants were analyzed for each treatment and this experiment was repeated three times with similar results. Photographs were taken at 30 dpi. **B** PCR analysis of ToLCNDV infection in tested melon cultivars. *Actin* was used as the internal control

To understand the resistance of the commercial melon cultivars to ToLCNDV, five commercial melon cultivars including Harukei, Xuelihong, Zhongkemi, Xizhoumi, and Dongfangmi were inoculated with the infectious clone of ToLCNDV-JS1. As shown in Fig. 5A, all the tested five melon cultivars displayed leaf curing symptoms. PCR amplification confirmed the presence of ToLCNDV infection in all the melon cultivars agroinoculated with the infectious clone ToLCNDV-JS1 (Fig. 5B). These results indicated that the selected five melon cultivars were susceptible to ToLCNDV.

## Discussion

ToLCNDV was first described infecting tomato in India in 1995. In recent years the virus is rapidly spreading and is widely distributed in Europe and Asia. In China, ToLCNDV was initially reported to infect tomato in Zhejiang Province in 2022 (Li et al. 2022). In this study, we characterized and analyzed the complete sequence of ToLCNDV isolate from Jiangsu Province of China. Sequence comparison and phylogenetic analysis showed that ToLCNDV-JS1 has the closest evolutionary relationship with the Zhejiang isolate of ToLCNDV in China.

Previous studies showed that ToLCNDV could infect a large number of economically important crop species belonging to 17 different families, such as *Cucurbitaceae* and *Solanaceae* (Seal et al. 2006). Infectivity assays showed that the infectious clone of ToLCNDV-JS1 constructed in this study is infectious and able to induce typical symptoms in *N. benthamiana*, melon, and watermelon plants, indicating the potential broad host range of ToLCNDV-JS1. Furthermore, viral progenies generated from melon plants inoculated with the infectious clone of ToLCNDV-JS1 are able to be mechanically transmitted to *N. benthamiana* plants and induce severe symptoms. Owing to the wide host range and multiple modes of transmission of ToLCNDV, more attention should be paid to the epidemiology of ToLCNDV and breeding of disease resistant cultivars.

Although previous studies reported that tomato cultivars harboring *Ty-2* and *Ty-3* or *SlSw5a* genes exhibited excellent resistance to ToLCNDV (Prasanna et al. 2015; Sharma et al. 2021), high susceptibility to ToLCNDV-JS1 was observed in all the five tested melon cultivars. The infectious clones of ToLCNDV constructed in this study provides a powerful tool for screening ToLCNDV resistant cultivars and investigate the functions of viral effectors through reverse genetic approaches.

## Materials and methods

### Plant materials and growth conditions

Melon plants showing leaf curling symptoms were collected from Jiangsu Province of China in October 2022. *N. benthamiana*, melon (*Citrullus melo*) and watermelon (*Citrullus lanatus*) plants used for virus inoculation were grown in an insect-free growth room at 25 °C under a 16 h light/8 h dark cycle.

### Total DNA extraction, PCR amplification, cloning, and sequencing

Total DNA was extracted from the leaves of field plants using CTAB buffer. Degenerate primer pair PA/PB was used to amplify part of the intergenic region (IR) and *AV2* gene of viral DNA (Zhou et al. 2001). The amplified PCR products were cloned into the pLB Vector (TIANGEN, Beijing, China) then sequenced. Based on the partial sequences determined, primer pairs ToLCNDV DNA A-1.0 mer BF/ToLCNDV DNA A-1.0 mer SR and ToLCNDV DNA B-1.0 mer BF/ToLCNDV DNA B-1.0 mer AR were designed and used to amplify the complete genome of ToLCNDV DNA A and DNA B, respectively. PCR products were purified and cloned into the pLB Vector (TIANGEN, Beijing, China), and sequenced by Sanger sequencing. Sequences were edited and assembled using Lasergene 7.0 (Madison, WI, USA). The sequences of primers used for PCR and cloning are shown in Table S1.

### Sequence analysis

The complete genome sequence of ToLCNDV-JS1 DNA A and DNA B was individually compared with known viruses available in the GenBank database using the BLASTn program. Phylogenetic analysis was performed using the full-length DNA A and DNA B of ToLNDV-JS1. A total of 445 begomovirus full-length sequences for the DNA A component and 155 begomovirus full-length sequences for the DNA B component were selected and used for phylogenetic analysis. Sequences were aligned by MAFFT (Katoh et al. 2002) using default settings and FASTA output and then automated alignment trimming by trimAL (Capella-Gutierrez et al. 2009) on LINUX operating system. Phylogenetic trees were constructed using neighbor-joining methods implemented in the MEGA 11 software (Tamura et al. 2021). 20 begomoviruses that share the closest relationship to ToLCNDV-JS1 were shown in the phylogenetic tree. The branches of the tree were bootstrapped with 1000 replicates.

### Construction of the infectious clone of ToLCNDV-JS1

To construct the infectious clone of ToLCNDV-JS1 DNA A, the full-length fragment of DNA A was amplified using primers ToLCNDV DNA A-1.0 mer BF/ToLCNDV DNA A-1.0 mer SR and cloned into pLB (TIANGEN, Beijing, China) to yield pLB-ToLCNDV DNA A. The clone pLB-ToLCNDV DNA A was digested with *Bam*HI and *Eco*RI to yield approximate 1.1 kb fragment encompassing the common region (CR) of ToLCNDV DNA A, and the product was introduced into the binary vector pBinplus to yield pBinplus-ToLCNDV DNA A-0.4mer. Then, the pLB-ToLCNDV DNA A was digested with *Bam*HI and *Sal*I to obtain a full-length fragment of ToLCNDV DNA A, which was then inserted into the unique *Bam*HI and *Sal*I site of pBinplus-ToLCNDV DNA A-0.4mer to generate pBinplus-ToLCNDV DNA A-1.4mer, containing a 1.4-mer tandem repeat ToLCNDV of DNA A. To generate the infectious clone of ToLCNDV-JS1 DNA B, the full-length genome of ToLCNDV-JS1 DNA B was amplified using primers ToLCNDV DNA B-BF/ToLCNDV DNA B-AR. The amplified fragment was cloned into pLB (TIANGEN) to yield pLB-ToLCNDV DNA B 1.0 mer. The clone pLB-ToLCNDV DNA B 1.0 mer was digested with *Bam*HI and *Eco*RI to yield an approximate 1.8-kb fragment, and inserted into the *Bam*HI and *Eco*RI sites of the binary vector pBinplus to yield pBinplus-ToLCNDV DNA A-0.4mer. pLB-ToLCNDV DNA B 1.0 mer was also digested with *Bam*HI and *Asc*I to yield the full-length fragment of ToLCNDV DNA B, which was then inserted into the unique *Bam*HI and *Asc*I site of pBinplus-ToLCNDV DNA B-0.7 mer to yield pBinplus-ToLCNDV DNA B-1.7 mer, containing a 1.7-mer tandem repeat of ToLCNDV DNA B.

All the constructs were sequenced. The resultant pBinplus-ToLCNDV DNA A-1.4mer and pBinplus-ToLCNDV DNA B-1.7 mer constructs were mobilized into the *Agrobacterium tumefaciens* strain EHA105 by electroporation.

### Agroinfection assays in N. benthamiana, melon, and watermelon plants

*A. tumefaciens* harboring the pBinplus-ToLCNDV DNA A-1.4mer or pBinplus-ToLCNDV DNA B-1.7 mer construct were grown individually. The cultures were collected, re-suspended with the induction buffer containing 10 mM MgCl_2_, 100 mM MES (pH 5.7), 2 mM acetosyringone, and kept at room temperature for 3 h. The suspensions were adjusted to OD_600_ = 1.0 before agroinoculation. Equal volume of agrobacteria culture harboring pBinplus-ToLCNDV DNA A-1.4mer and pBinplus-ToLCNDV DNA B-1.7 mer were mixed and infiltrated into leaves of *N. benthamiana*, melon, or watermelon plants using 1 mL needleless syringes as described (Mei et al. 2021). Inoculated plants were grown in an insect-free greenhouse and monitored for symptom development.

### Mechanical transmission of ToLCNDV

The systemic leaves of ToLCNDV-infected melon (0.5 g) were homogenized in 5 mL of 0.01 M PBS buffer. The crude sap was gently rubbed onto celite-dusted surface of four to six-leaf stage *N. benthamiana* plant leaves. *N. benthamiana* plants mechanically inoculated with the crude sap of healthy melon leaves served as the mock control. Inoculated *N. benthamiana* plants were grown in an insect-free growth room at 25 °C under a 16 h light/8 h dark cycle. Samples used for immunoblot analysis was collected at 9 dpi.

### Protein extraction and immunoblot analysis

Plant leaf tissues (0.1 g) were harvested, ground to a fine powder in liquid nitrogen, and mixed with a ratio of 1:1 of protein extraction buffer (50 mM Tris–HCl, pH 6.8, 4.5% SDS, 9 M Urea) supplemented with protease inhibitor cocktail (MedChemexpress, NJ, USA). The homogenate was centrifuged at 8,000 g for 10 min, and the supernatant were separated by 12.5% SDS-PAGE for immunoblot analysis using indicated antibodies.

### Supplementary Information

**Additional file 1: Table S1.** Primers used in this study.

## Data Availability

The data that support the findings of this study and the materials used during the current study are available from the corresponding author on reasonable request.
