# Supplementary material for: Molecular characterization and pathogenicity of an infectious clone of tomato leaf curl New Delhi virus isolate infecting Cucumis melo
Source: Stress Biol. 2023 Nov 23;3(1):51. doi: 10.1007/s44154-023-00128-8 (PMC10667179; doi:10.1007/s44154-023-00128-8)
Supplement: Supplementary file 1 — Additional file 1: Table S1. Primers used in this study. [file 44154_2023_128_MOESM1_ESM.docx]

**Table S1 Primers used in this study**

| Primer name | Primer sequences |
| --- | --- |
| ToLCNDV DNA A-1.0 mer BF | GGATCCACAAACATGTGGGATC |
| ToLCNDV DNA A-1.0 mer SR | GTCGACGGATCCAAACTTGGTGAGCAAGTCTATTTATTGA |
| ToLCNDV DNA B-BF | GGATCCAAACATTTCAACAAAAGG |
| ToLCNDV DNA B-AR | GGCGCGCCGGATCCAAACATTTCAACAAAAGGTATTAGCGGT |
| PA | TAATATTACCKGWKGVCCSC |
| PB | TGGACYTTRCAWGGBCCTTCACA |
| ToLCNDV DNA A-Detection-F | GTCCATCGATCTGAAAACACC |
| ToLCNDV DNA A-Detection-R | CAAACCCTAGAAACCCCAACG |
| ToLCNDV DNA B-Detection-F | ACTAACAGACTTTATGGAAAT |
| ToLCNDV DNA B-Detection-R | GCATAAAATTCAAACAAGTATTG |
